# Clinical Society of New York Polyclinic Medical School and Hospital

**Published:** 1912-05

**Authors:** 


For Texas Medical Journal.

### Clinical Society of New York Polyclinic Medical School and Hospital.

Meeting of March 4, 1912.

### Case of Foreign Body in the Eye; X-Ray Localization; General Infection of the Eye Controlled by Urotropin.

CASE PRESENTED BY DR. EARL CONNER.

The patient, a young man of 22, called upon Dr. Conner, with a history of being employed in hammering or using a chisel on a piece of cold steel. A piece of the metal flew and struck him in the eye. When seen four days later the eye was swollen, and painful; the anterior chamber was filled with pus, and on the nasal side of the globe there was a minute punctured wound, about 4 mm. in size. The patient had not slept the night before. He was informed that the eye was infected and that in all probability would be lost, though every effort would be made to save it.

On admission to the hospital, the test was made with the giant magnet. The result, however, was neither positive or negative. If there is a foreign body in the eye, it is apt to give pain on being brought into the field of the magnet. In this case, the temporal side of the eye was brought into the field of the magnet, and the patient experienced sharp pain, but, after a dozen trials, no foreign body could be located at any point. The patient was put to bed, given atropine, and hot applications of calomel.

The pupil was dilated and pus in the anterior chamber absorbed. and the interior of the eye examined. A yellow reflection was obtained, which was evidence of infection of the vitreous. The inflammation increased from day to day, up to the tenth day before it was possible to control it. After the patient was put to bed he was given calomel, followed later by seven and a half grains of urotropin three times a day for two weeks. This drug has been administered in a large number of cases of eye infection with very favorable results. Dr. Conner said that he had had three cases of eye infection in the past few weeks in which the infection had apparently been controlled by the use of urotropin.

Skiagraphs had been made of the eye for the purpose of determining the presence or absence of a foreign body. The size of the foreign body as determined by the skiagraph was 1 mm. by 1½ mm. As a rule, these cases go on to the loss of the eye. An infection of the vitreous is seldom or never arrested. The usual termination is perforation of the eye and hemorrhage. The inflammation in this instance had been controlled, the pain relieved, the patient has perception of light, and there is hope of saving the eye.

### Operation Subsequent to Gillian Operation.

#### TWO CASES PRESENTED BY DR. C. G. CHILD.

Dr. Child reported two cases operated on by the Gillian method which had returned after being operated upon. He said that neither case had been relieved by this method, and were suffering from pelvic pain with dragging on the abdominal wall, and this in spite that both operations had been done by most competent men.

Dr. Child said that he objected to the operation, as the intestines and omentum later became incarcerated in the pelvis. It produced pathological conditions which were worse than the original displacement of the uterus. Varicose conditions followed, and an even worse state of things supervened in the formation of pockets in the pelvis.

In both of the cases he reported, pockets had formed, in one of which the sigmoid was very extensively adherent, and constipation had been obstinate.

Dr. Tovey said that Dr. Wells used to do the operation until he found that the patients came back with the uterus in the same position, and all the troubles increased, or else they complained of pain in the side where the ligaments were attached. They had abandoned the operation.

### Hematuria in a Multipara; Unknown Case.

#### PRESENTED BY DR. WARD B. HOAG.

Dr. Hoag reported a case of hematuria in a multipara forty years of age, in her fourth pregnancy. At six and a half months she developed, without any discomfit a considerable quantity of blood in the urine. Beyond the presence of blood, there was no pain or discomfit of any kind.

The patient was put to bed, irrigations of alum solution used, and rest enjoined for ten days. It had no effect on the bleeding. She went on to full term and was delivered in a perfectly normal way. The hemorrhage continued for two weeks after the child was born, and then stopped as suddenly as it had begun. Dr. Hoag thought that it was the result of intra-abdominal pressure, perhaps the rupture of a small blood-vessel. The same night there was a little fleshy plug passed in the urine which was the only thing ever seen, and coincident with this the hemorrhage stopped and has not since returned.

Dr. Shears said that, although the hemorrhage might be due to toxic conditions, in the present instance there were no signs of a toxemia. Ruling out local papilloma or a ureteritis, he thought that six and a half or seven months was not too early to exert pressure symptoms sufficiently severe to produce hemorrhage. From a careful examination of the bladder, and the absence of stone or other aggravating cause, he should be inclined to attribute the bleeding to this cause.

